# The Effect of Massage on Anticipatory Anxiety and Procedural Pain in Patients with Burn Injury

**Published:** 2017-01

**Authors:** Tahereh Najafi Ghezeljeh, Fatimah Mohades Ardebili, Forough Rafii, Farzad Manafi

**Affiliations:** 1 Department of Critical Care Nursing, School of Nursing and Midwifery, Iran University of Medical Sciences, Tehran, Iran;; 2 Department of Medical-Surgical Nursing, Faculty of Nursing and Midwifery, Iran University of Medical Sciences, Tehran, Iran;; 3 Center for Nursing Care Research, Department of Medical-Surgical Nursing, School of Nursing and Midwifery, Iran University of Medical Sciences, Tehran, Iran;; 4 School of Medicine, Iran University of Medical Sciences, Tehran, Iran

**Keywords:** Anticipatory anxiety, Burn, Massage, Pain

## Abstract

**BACKGROUND:**

Pain related to burn injuries is one of the most troublesome pain intensity. This study aimed to investigate the effect of massage on anticipatory anxiety, procedural pain intensity, vital signs and relaxation level of patients with burn injury.

**METHODS:**

In this quasi-experimental study, through convenience sampling, 60 hospitalized adult burn patients were selected from a specialized burn and reconstructive hospital. Subjects were assigned to massage and control groups through simple randomization. Massage was offered by using non aromatic oil about 10-15 minutes before wound care on intact part of the body once a day for 20 minutes on patients’ bedside for 3 consecutive days. In the 3 days, the control group did not received any massage and were asked to stay at bed. Demographic and clinical characteristics and vital signs, Visual Analogue Scale and the Persian version of Burn Specific Pain Anxiety Scale were used to determine baseline and procedural pain, anxiety and relaxation levels and anticipatory anxiety.

**RESULTS:**

No significant difference was noted between mean score of pain intensity, anxiety and relaxation level, and vital signs in massage and control groups after intervention following wound care. In massage and control groups, there was no significant differences between mean scores of anticipatory anxiety before and after intervention. There was no significant difference between the mean scores of anticipatory anxiety in massage and control groups after intervention prior wound care.

**CONCLUSION:**

Massage was shown not to have any effect on anticipatory anxiety and procedural pain.

## INTRODUCTION

Burn injury is a major cause of morbidity and mortality imposing a high cost in health care system. After burn injuries, patients encounter variety of painful procedures in the treatment course.^1^ For promoting healing, burn patients should have wound care, including removing dressing, cleaning the wound, debridement, applying antiseptic ointments and dressing again,^2^ while most patients report excruciating pain during this procedure.^3^ Patients describe procedural burn pain as scaring and they may have negative reactions to painful procedures.^4^

Also the anticipation of painful procedures can lead to anxiety and fear. Pain related to procedures and its anticipatory anxiety result in detrimental impact on patients including fears, post-traumatic stress, metabolic and immunologic disturbances,^5^ and sleep problems.^6^ Also it can interfere with their compliance and result in less trust to health care providers and not cooperating with them.^1^ Finally, it would cause delay in wound healing and recovery process. As a result, effective strategies should be considered for reducing anxiety, distress and pain related to burn wound care for improving the overall well-being of patients.

The primary choice of relieving pain and anxiety in burn patients are opioids. Repeated administration of opioids can result in tolerance and increasing doses of opioids should be done to achieve effective relief. Also it can result in physical dependence.^7^ Low doses of opioids are not effective and with higher doses, side effects become progressively troublesome. Complementary therapies may use in addition to analgesics to decrease procedural pain and its related anxiety.^8^

Massage is a common complementary therapy that often has been applied with inadequate evidence for its efficacy.^9^ It is a simple technique for managing pain through different ways, including induction of physical and psychological relaxation responses, increased blood flow and parasympathetic activity,^10^ releasing endorphins, decreasing inflammation, secretion of catecholamine, oxygen consumption, muscle spasm,^11^ closing the gate of pain,^12^ and promoting a feeling of well-being and receiving good care.^13^ Massage may improve the function of immune system too.^14^ Nurses should investigate the effects of different non-pharmacological interventions on pain.^15^ Further researches are necessary prior to massage become mainstream intervention for pain and anxiety relief. 

Limited studies have evaluated the benefits of massage prior to burn wound care in reducing procedural pain and anticipatory anxiety. However, the effect of jaw relaxation before wound care on pain-related anxiety in burn patients was investigated and significantly reported less pain-related anxiety after intervention and before wound care.^16^ Investigating this issue could provide evidence regarding the possible utilization of non-pharmacological interventions during wound care of burn patients.^17^ Field *et al.*^18^ reported that massage can reduce pursuits, pain and psychological symptoms after burn injury. Hernandez-Reif *et al.*^19^ noted that massage in burn patients can increase pain threshold and patients tolerance for procedural pain. Although some studies of massage indicated that it may have useful effects in patients with various conditions, but methodological issues in these studies limit their internal validity and generalizability.^9^^,^^20^^,^^21^ The other reason for these compromises is that research regarding complementary therapies and symptom management often have less financial supports,^22^^,^^23^ even though symptom management is critical for the patient’s quality of life.^24^

This study aimed to investigate the effect of massage that implemented before wound care on anticipatory anxiety, procedural pain intensity, vital signs and relaxation level. It was hypothesized that compared to control group, burn patients who received massage would have a greater decrease in anticipatory anxiety; reduction in procedural pain intensity; decrease in blood pressure and pulse rate; reduction in anxiety level and increase in relaxation level. 

## MATERIALS AND METHODS

This study was a quasi-experimental, pretest-posttest comparison group design. Ethical approval was granted from Research Ethical Committee of Tehran University of Medical Sciences. Confidentiality and anonymity were guaranteed throughout the study. Informed consent form was signed by all participants and they also gave verbal agreement to receive massage before each session.

This study was carried out in Motahari Burn and Reconstructive Center affiliated to Iran University of Medical Sciences in Tehran, Iran. The sample size was estimated 30 subjects for each massage and control groups with medium effect size (ES) of 0.65, power 85%, an alpha of .05, and 10% drop-out rate. The homogeneity of groups was assessed regarding demographic and clinical variables. Burn patients were asked to participate in this study between November 2013 and March 2014. Inclusion criteria of participants were 1) age older than 18 years; 2) having no cognitive disorder; 3) at least 72 hrs. passed from burn, 4) patients with severe burn injury (total body surface area (TBSA) more than 20%) and 5) to be able to communicate verbally. The exclusion criteria were deterioration of patient’s condition and not receiving intervention in all 3 sessions of the study.

Through convenience sampling, 60 patients were recruited. After obtaining informed consent, participants were allocated to massage and control groups based on simple randomization method. Blinding participants, massage therapists, or researcher was not possible because of the nature of intervention; however, a co-researcher who conducted data gathering and entry was blind about the groups. 

The 3 researcher-designed forms were considered to document demographic and clinical characteristics, and vital signs (pulse, systolic and diastolic blood pressure and respiration rate). In 1^st^ day before intervention, demographic and clinical characteristics of patients in both massage and control groups were determined by a brief interview and use of medical records. Demographic characteristics included age, gender, marital status, educational level, adequacy of income, ethnicity and occupation status. Clinical characteristics included history of hospitalization due to burn injury, cause of burn, TBSA, burn degree, and burn parts of the body. 

In 3 consecutive days after intervention and wound care, vital signs (brachial pulse rate, respiration rate, and systolic and diastolic blood pressures) of patients in two groups were measured and documented by co-researcher. The brachial pulse and respiration rates were counted for a minute in a comfortable position for patient. Blood pressure was monitored by portable analog sphygmomanometer through auscultation. 

Behavioral Pain Scale to assess pain (BSPAS) is a self-reporting scale and measures anticipatory anxiety in burn patients experiencing painful procedures.^25^ High internal consistency and good validity of Persian version of BSPAS has been reported.^26^ BSPAS has 9 items; each including a visual analogue scale (VAS), with digits ranging from 0 (not at all) to 10 (the worst imaginable way). The patients were asked to rate VAS for each item, then the total score was calculated by summing the scores for all items. The range of the scores was 0–90. The higher the value rated, the anticipatory anxiety level was greater. Anticipatory anxiety should be measured before painful procedures including wound care.^8^ In current study, in 3 consecutive days before wound care and intervention, and about 10-15 minutes after intervention, the patients were asked to fill the scale to measure anticipatory anxiety.

Wound care procedures were long lasting with fluctuations in pain. Its related pain intensity and distress was measured following wound care to have accurate measurement.^8^ The VAS was a reliable and valid scale to measure subjective information including pain intensity and anxiety level.^27^^,^^28^ After intervention and wound care, patients in both groups were asked to rate their pain intensity on a VAS, ranging from 0 (no pain/discomfort) to 100 (the worst pain). The higher the value rated, the greater the pain intensity was. Also patients were asked to rate their relaxation levels at that point of time on a VAS, ranging from 0 (quiet relax) to 100 (no relaxation). The higher the value rated, the relaxation level was lower. A separate VAS, ranging from 0 (no anxiety) to 100 (highest anxiety level), were used for assessing anxiety among patients in both groups. The higher the value rated, the greater the anxiety level was.

Massage was offered on intact part of the body once a day for 20 minutes on patients’ bedside for 3 consecutive days. Burned part of the body had dressing and 2 co-researchers who were certified massage therapists performed massage for the patients. By coordinating with staff, using paravan and closing the door, co-researchers facilitated patients’ relaxing during intervention. Participants were draped with a sheet over the body and only the needed portions of the body for massage were exposed. 

Swedish massage (including effleurage, petrissage, tapotement, friction and vibration) was performed by using non-aromatic oil about 10-15 minutes before wound care. The therapists performed massage in a comfortable position for patient from shoulders to toes and vice versa on only intact portions of the body and within one inch of dressing. They applied massage in the direction of the patients’ heart. During massage intervention, the speed, depth and pressure of the hands on body were tried to be stable. In 3 days, the control group did not receive massage and were asked to stay at bed. 

Data was analyzed using SPSS software (Version 20.0, Chicago, IL, USA). A P≤0.05 was considered statistically significant. The average score of outcome measures in three days was considered for data analysis. All Data was normally distributed. Patients’ baseline demographic and clinical characteristics were summarized using descriptive statistics. Between-group differences at baseline were investigated using Chi-square and Fisher’s Exact test for categorical data and independent t-test for continuous data. Paired t-test was used to compare pain, anxiety and relaxation levels, and vital sign levels within groups. Mean scores of changes in these variables before and after intervention was considered for comparing groups. 

## RESULTS

Sixty burn patients participated in the study (30 patients in each groups), with dropout rates of 0% and response rate of 100%. Patients’ demographic and clinical characteristics were presented in Table 1. Patients’ demographics and clinical characteristics were similar in massage and control groups in terms of age, sex, marital status, job, economical status and ethnic group and cause, region, degree and percentage of burn injury. The majority of participants were married, employed, at moderate economical status and ethnic groups. In massage group, most participants were male with diploma degree. 

**Table 1:** Demographic and clinical characteristics of burn patients in massage and control groups (n=60).

**Groups ** **Characteristics** **No (%)**		**Overall**	**Massage** **(n=30)**	**Control** **(n=30)**	**Test result**	**Level of significance**
		**No (%)**	**No (%)**			
**Demographic **						
Sex	Female Male	30 (50.00) 30 (50.00)	13 (43.30) 17 (56.70)	17 (56.70) 13 (43.30)	1.06	0.30**
Marital status	Single Married Divorce	18 (30.00) 37 (61.70) 5 (8.30)	12 (40.00) 17 (56.70) 1 (3.30)	6 (20.00) 20 (66.70) 4 (13.40)	4.58	0.21***
Job	Nonemployee Employee Housekeeper	8 (13.30) 35 (58.30) 17 (28.30)	7 (23.30) 16 (53.30) 7 (23.30)	1 (3.30) 19 (63.30) 10 (33.30)	3.36	0.07***
Educational level	Illiterate Elementary High school Diploma University degree	5 (8.30) 13 (21.70) 14 (23.30) 17 (28.30) 11 (18.30)	2 (6.70) 6 (20.00) 5 (16.70) 11 (36.70) 6 (20.00)	3 (10.00) 7 (23.30) 9 (30.00) 6 (20.00) 5 (16.70)	1.09	0.29***
Economic status	Good Moderate Weak	9 (15.00) 35 (58.30) 16 (26.70)	3 (10.00) 20 (66.70) 7 (23.30)	6 (20.00) 15 (50.00) 9 (30.00)	0.04	0.84***
Ethnical group	Fars Kurd Turk Baloch Lor	43 (71.70) 2 (3.30) 12 (20.00) 1 (1.70) 2 (3.30)	22 (73.30) 0 (0.00) 6 (20.00) 1 (3.30) 1 (3.30)	21 (70.00) 2 (7.70) 6 (20.00) 0 (0.00) 1 (3.30)	0.014	0.90***
Age, yr., mean (SD), (95% CI)		32.90 (8.58), (30.68, 35.12)	31.23 (8.69), (27.99, 34.48)	31.25 (8.11), (31.47, 37.66)	1.52	0.13*
*Clinical *						
Burn reason	Self-inflect Event Criminal	4 (6.70) 54 (90.00) 2 (3.30)	3 (10.00) 26 (86.70) 1 (3.30)	1 (3.30) 28 (93.30) 1 (3.30)	0.66	0.40**
Burn factor	Scald Flame Electricity Gas explosion	4 (6.70) 48 (80.00) 2 (3.30) 6 (10.00)	2 (6.70) 23 (76.70) 2 (6.70) 3 (10.00)	2 (6.70) 25 (83.30) 0 (0.00) 3 (10.00)	0.14	0.71***
Burn locations	Hand, yes Foot, yes Front of the body, yes Back, yes Head and face, yes	55 (91.70) 54 (90.00) 47 (78.30) 18 (30.00) 40 (66.70)	28 (93.30) 26 (86.70) 21 (70.00) 11 (36.70) 22 (73.30)	27 (90.00) 28 (93.30) 26 (86.70) 7 (23.30) 18 (60.00)	0.22 0.73 2.41 1.25 1.18	0.64*** 0.39*** 0.12*** 0.26*** 0.28***
Burn degree	1-2 2 2-3 1-2-3	8 (13.00) 7 (11.70) 25 (41.70) 20 (33.30)	3 (10.00) 1 (3.30) 15 (50.00) 11 (36.70)	5 (16.70) 6 (20.00) 10 (33.30) 9 (30.00)	2.78	0.10***
TBSA a, mean (SD), (95% CI)		32.57 (8.18), (30.45, 34.68)	33.00 (8.96), (29.76, 36.44)	32.03 (8.17) (29.26, 34.81)	0.50	0.62*

* Independent t-test;

** Chi-Square test;

*** Fisher exact test;

a Total Body Surface Area

In control group, most of them were female with high school level of education. The mean age of participants was 32.90 (8.58) years. All in both groups were pre-medicated for pain related to wound care. In massage and control groups, the majority of participants experienced flame 2^nd^ to 3^rd^ degree burns. In all patients, more than two body areas had burn injuries. The mean TBSA was 33.00% (8.96) and 32.03% (8.17) in massage and control groups, respectively. Also patients’ baseline characteristics before intervention were similar in massage and control groups (Table 2).

**Table 2:** Mean score of baseline characteristics of burn patients in massage and control groups (n=60).

**Groups ** **Baseline Characteristics **	**Massage (n=30)**		**Control (n=30)**		**Test result**	**P value**
	**Mean (SD)**	**95%CI**	**Mean (SD)**	**95%CI**		
Pain intensity	89.33 (18.88)	74.25, 90.08	82.17 (21.20)	82.28, 96.38	1.38	0.17
Anxiety level	91.00 (18.68)	80.59, 97.07	88.83 (22.08)	84.02, 97.97	0.41	0.68
Relaxation level	91.50 (17.43)	80.68, 96.99	88.83 (21.84)	84.99, 98.88	0.52	0.60

The mean procedural pain intensity, and level of anxiety, relaxation and vital signs in massage and control groups were demonstrated in Table 3 indicating no significant difference between mean score of pain intensity, anxiety level, respiration rate, and systolic and diastolic blood pressure between massage and control groups after intervention following wound care. Also, according to Mann-Whitney test, there was not any statistically significant difference between relaxation level and pulse rate of massage and control groups after intervention following wound care. 

**Table 3:** Mean scores of procedural pain intensity, and level of anxiety, relaxation and vital signs in massage and control groups (n=60).

**Variables**	**Massage (N=30)**		**Control (N=30)**		**Significance**
	**Mean (SD)**	**95% CI**	**Mean (SD)**	**95% CI**	
Pain intensity	70.72 (26.96)	60.65, 80.79	81.66 (30.98)	70.09, 93.23	t=-1.45, P=0.15
Anxiety level	64.89 (28.46)	54.26, 75.52	78.72 (32.39)	66.62, 90.81	t=-1.76, P=0.08
Relaxation level	75.05 (21.78)	66.92, 83.19	77.38 (33.77)	64.78, 89.99	Z=-0.32, P=0.75*
Pulse	79.02 (5.65)	76.91, 81.13	81.15 (7.68)	78.29, 84.02	Z=-2.59, P=0.06*
Respiration	23.88 (3.47)	22.58, 25.17	24.08 (5.29)	22.10, 26.05	t=-0.17, P=0.86
Systolic blood pressure	122.80 (35.77)	109.43, 136.16	119.37 (8.98)	116.02, 122.73	t=0.51, P=0.61
Diastolic blood pressure	67.04 (3.82)	65.62, 68.47	68.02 (4.5)	66.32, 69.73	t=-0.90, P=0.37

* Mann-Whitney

The mean anticipatory anxiety in both massage and control groups awere summarized in Table 4. The findings revealed that burn patients in both groups experienced high levels of anticipatory anxiety before intervention. In massage group, there was no statistically significant difference between mean scores of anticipatory anxiety before and after intervention. Also, the results of paired t-test showed no significant difference between mean of anticipatory anxiety, before and after intervention in control group. The independent t-test indicated that there was no significant difference between mean of anticipatory anxiety before intervention in massage and control groups. Furthermore, there was no significant difference between the mean scores of anticipatory anxiety in massage and control groups after intervention.

**Table 4:** Mean scores of anticipatory anxiety in massage and control groups (n=60).

**Groups**		**Massage (N=30)**		**Control (N=30)**		**Significance** *
**Variables**	**Time**	**Mean (SD)**	**95%CI**	**Mean (SD)**	**95%CI**	
Anticipatory anxiety	Before intervention	80.33 (17.05)	73.96, 86.69	79.86 (18.26)	73.04, 86.69	t=0.102, P=0.92
	After intervention before wound care	80.31 (17.04)	73.96, 86.68	79.85 (18.86)	73.04, 86.68	t=0.101, P=0.92
	Significance **	t=-1.00, P=0.33		t=-1.43, P=0.16		

* Independent t-test;

** Paired t-test

## DISCUSSION

In this study, we assessed the effect of massage on anticipatory anxiety, procedural burn pain intensity and anxiety and relaxation level showing that massage did not decrease anticipatory anxiety and procedural pain anxiety level and vital signs among burned patients. The study hypotheses were rejected and indicated that compared to control group, burn patients who received massage did not have any decrease in anticipatory anxiety; procedural burn pain intensity; anxiety level; blood pressure; respiratory rate and pulse rate. The data suggest that massage did not increase in relaxation level in experimental group compared with control group. 

The study findings indicated that massage did not decrease anticipatory anxiety due to wound care in burn patients. Inconsistent with current results, Field^29^ reported that massage before or during treatment reduced anxiety and pruritus and increased relaxation level in children with burns and eczema. Hernandez-Reif *et al.*^19^ reported that burn children in a massage group indicated less distress behaviors and movement during wound care as compared with control group. Also nurses reported that wound care for children in experimental group was easier than control group. 

The differences between the results of current study with previous research can be related to different research population. The literature mostly focused on burn children and in current study on burn patients who were adults. They may differ in experience and perception of and response to distress related to wound care. Also Field *et al.*^30^ indicated that 30 minutes massage for 5 days before debridement reduced anxiety and cortisol levels in burn patients. The difference may be related to the fact that in this study patients experienced severe burn injuries (TBSA more than 20%). As Hanafiah *et al.*^31^ mentioned that burn size, depth and location, anxiety and depression, inflammation, healing progression and grafting can influence pain intensity. 

Loncar *et al.*^32^ found significant relationship between TBSA and pain scores and illustrated that TBSA is a significant predictor of burn pain. Also they found that higher levels of anxiety and depression were related to higher pain scores. Because of bidirectional relation between pain and anxiety, there was no significant reduction in procedural pain intensity and anxiety level after wound care. Also massage did not continue in dressing room which can be another reason for not effecting on procedural pain, anxiety and relaxation and physiologic measure. Byers *et al.*^33^ indicated that during treatment anxiety and procedural pain, level of burn patients was higher than the baseline and background anxiety. However, it might be difficult to relax and stay stable for massage simultaneous with conducting wound care.

According to our findings, massage was not effective in managing anticipatory anxiety and procedural pain intensity in burn patients with severe injury. The data suggest that massage did not improve procedural anxiety and relaxation levels and physiologic measures among patients with burn. Any invasive procedure can produce pain and anticipatory anxiety. Not preventing and treating pain and anxiety may cause detrimental effects in burn patients. Therefore, more research is needed to investigate different complementary therapies for controlling procedural pain and anticipatory anxiety. Also nurses play important and paradoxical roles in managing pain in burn patients and it is essential to consider most effective approaches to manage it. 

In our study, random allocation was not conducted; however, homogeneity of the groups was tested. Therefore, it is recommended to replicate this study by considering random allocation too. In this study, the sample size was small, so it is recommended to conduct further investigation with larger sample sizes. In current study, Swedish massage was used and the results indicated no efficay. It is recommended to investigate and compare other techniques of massage to indicate the most effective one for improving anticipatory anxiety and procedural pain in burn patients. 

Also, in current research; 20 minute massage was performed 10 to 15 minutes before procedure. Since there is not standard time length for conducting massage, it is recommended to investigate different time intervals for massage to determine the effective time for improving patients’ symptoms. Also, there is not information about wash out period time of massage and perhaps conducting massage immediately before wound care and continuing it in dressing room can affect on anticipatory anxiety and procedural pain intensity. Therefore, further researches are recommended too. Because of the nature of the intervention, Hawthorne effect should also be considered in the study.
